# Safety and efficacy of high‐dose methotrexate for osteosarcoma in adolescents compared with young adults

**DOI:** 10.1002/cam4.1898

**Published:** 2018-12-22

**Authors:** Brittany Wippel, Kenneth R. Gundle, Theresa Dang, Jillian Paxton, Joseph Bubalo, Linda Stork, Rongwei Fu, Christopher W. Ryan, Lara E. Davis

**Affiliations:** ^1^ School of Medicine (SOM) Oregon Health & Science University Portland Oregon; ^2^ Department of Orthopedics & Rehabilitation, SOM Oregon Health & Science University Portland Oregon; ^3^ Operative Care Division Portland Veterans Affairs Medical Center Portland Oregon; ^4^ Knight Cancer Institute, SOM Oregon Health & Science University Portland Oregon; ^5^ Department of Pediatrics, SOM Oregon Health & Science University Portland Oregon; ^6^ School of Public Health Oregon Health & Science University Portland Oregon

**Keywords:** adolescents and young adults, efficacy, methotrexate, osteosarcoma, toxicity

## Abstract

**Background:**

Doxorubicin, cisplatin, and high‐dose methotrexate (HDMTX) are the backbone of pediatric osteosarcoma treatment. However, due to toxicity concerns and the lack of data regarding efficacy in adults, high‐dose methotrexate is rarely used in the adult population.

**Methods:**

This single‐center retrospective study examined 33 patients who received HDMTX (12 g/m^2^, maximum 20 g) for the treatment of osteosarcoma at Oregon Health and Science University (OHSU) from 2011 to 2017. Time to serum methotrexate level ≤0.1 µmol/L was the primary outcome. Secondary outcomes included number of HDMTX doses received, methotrexate‐related toxicities, and disease outcomes including histologic response at resection and metastasis‐free survival.

**Results:**

Median age was 20 years [range 7‐38]; 14 patients ≤18 years old and 19 patients >18 years old. Median time to clearance for patients ≤18 years was 79 hours (range 63‐116) compared to 120 hours (range 77‐315) for patients >18 years (*P* < 0.001). No correlation between age and histologic response at resection was observed (*P* = 0.50), but there was a significant positive correlation between the number of HDMTX doses received before resection and histologic response (*r* = 0.49, *P* = 0.006). There was no significant difference in metastasis‐free survival between age groups, although a trend toward improved survival was noted for patients who received at least seven doses of HDMTX.

**Conclusion:**

Age over 18 years correlates with delayed methotrexate clearance and fewer administered doses of methotrexate, without increased toxicity. The potential benefit of HDMTX in young adults with osteosarcoma may outweigh toxicity risks.

## 1 INTRODUCTION

### 1.1 Background

Osteosarcoma is the most common primary bone tumor and is among the most commonly diagnosed tumors in adolescents and young adults (AYA). The peak incidence of osteosarcoma occurs during late adolescence with a second peak occurring in the elderly.^1^ Outcomes for young adults with osteosarcoma are worse than for pediatric patients, but the reason for the increased rate of relapse is unclear.^2^

Doxorubicin, cisplatin, and high‐dose methotrexate (HDMTX) have been the backbone for treatment of osteosarcoma in the pediatric setting for decades.^1^, ^3^, ^5^ HDMTX for children with osteosarcoma is standard of care throughout the United States and Europe, but is uncommonly used in adults, presumably due to concerns over toxicity and skepticism regarding efficacy. Methotrexate can be associated with life‐threatening complications, and there is significant variability of methotrexate pharmacokinetics, making clearance difficult to predict.^6^ Prior research has shown that 24‐hour serum concentrations are higher in adults, leading to a greater risk for toxicity and longer time to clearance.^7^ Delayed clearance can also lead to decreased dose intensity of doxorubicin and cisplatin.^8^ Combined with the fact that studies on the efficacy of methotrexate were done in younger populations, providers are hesitant to use HDMTX in patients over the age of 18 who are treated at adult medical centers.^6^ Nonetheless, curative‐intent osteosarcoma drug regimens that include methotrexate may result in significantly better event‐free and overall survival.^9^

### 1.2 Objectives

We hypothesized that young adult patients ages 19‐40 may have delayed clearance of HDMTX compared to patients 18 years old and younger, and that prolonged time to clearance leads to decreased number of HDMTX doses received.

## 2 METHODS

### 2.1 Setting

We conducted a retrospective chart review of patients who received HDMTX for the treatment of osteosarcoma at Oregon Health and Science University (OHSU) from 1 January 2011 to 31 July 2017. Generally, patients under the age of 18 years were treated at OHSU Doernbecher Children's Hospital (DCH) and patients over 18 years old were treated at OHSU Knight Cancer Institute (KCI), although 18 year olds were treated at either location.

### 2.2 Participants and study size

**Figure 1:**
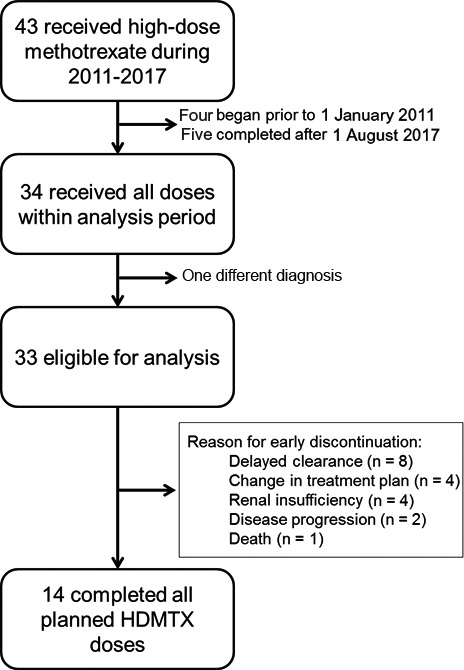
Study diagram

Following institutional review board approval, pharmacy administration records identified 43 patients treated with methotrexate at a dose of 12 g/m^2^ (maximum 20 g) during the study period. Eligible subjects must have completed all doses of HDMTX within the study period and have been treated for a diagnosis of osteosarcoma. One patient was excluded for having a transformed giant‐cell tumor of bone, and nine were excluded for receiving HDMTX outside of the given study period. Thirty‐three patients were ultimately eligible for inclusion (Figure 1). For the purposes of this study, adolescents were defined as ages ≤18 years and young adults as >18 years.

### 2.3 Data sources and quantitative variables

HDMTX was administered in the inpatient setting as a 4‐hour infusion, followed by leucovorin 15 mg/m^2^/dose beginning at hour 24 and continuing every 6 hours until documented clearance of methotrexate from the serum, defined as a concentration of ≤0.1 µmol/L by immunoassay. Intravenous hydration was standardly given at a rate of at least 200 mL/h (125 mL/m^2^/h for children) with alkaline fluids to facilitate urinary methotrexate excretion. Adjustments to leucovorin dose and frequency and fluid rate were based on methotrexate serum levels and generally followed the supportive care recommendations of Children's Oncology Group (COG) clinical trial protocols.^10^ Patients were discharged only after reaching a serum methotrexate concentration of ≤0.1 µmol/L.

The primary outcome was HDMTX clearance time, defined as time of HDMTX infusion start to time to first serum methotrexate level ≤0.1 µmol/L. Difference in clearance time between the two study groups (≤18 years vs >18 years) was determined in the primary analysis. Secondary outcomes included number of HDMTX doses received and methotrexate‐related toxicities, assessed by age and setting (pediatric vs adult inpatient units), as well as disease‐specific outcomes of histologic response at resection per clinical pathology report and metastasis‐free survival.

Toxicity was defined as hospitalization beyond methotrexate clearance, renal insufficiency (serum creatinine above the upper limit of normal [ULN] for patients ≥18 years, or >50% increase from baseline for patients <18 years during any one HDMTX cycle), or readmission for adverse events identified as methotrexate‐related. Long‐term impact on renal function was assessed by comparing estimated glomerular filtration rate (eGFR) at baseline (prior to the start of chemotherapy) and treatment end (after last dose of HDMTX). eGFR was calculated using age‐appropriate MDRD online calculators from the National Kidney Foundation (https://www.kidney.org/professionals/KDOQI/gfr_calculatorPed and https://www.kidney.org/professionals/kdoqi/gfr_calculator).

Metastasis‐free survival was determined based on the time from diagnosis until detection of recurrent disease or final encounter during the study time period. Patients with metastases at diagnosis were excluded from these analyses. Patients who did not have an event (metastasis or death) or were lost to follow‐up were considered censored at last encounter. All outcomes and diagnostic criteria were clearly defined to reduce experimenter bias.

### 2.4 Statistical methods

Continuous and categorical variables were compared between groups by Wilcoxon rank sum test and Fisher's exact test as appropriate, and correlations were assessed with the Spearman correlation coefficient. Due to the sample size and non‐normality of some variables, nonparametric tests were favored; however, parametric equivalents were calculated without any meaningful change in results. Categorical outcomes were compared by Fisher's exact test. No multivariate analyses were completed. Metastasis‐free survival was analyzed using the Kaplan‐Meier estimator and compared using the log‐rank test. Statistical analyses were performed in R version 3.4.1 (Vienna, Austria).

## 3 RESULTS

### 3.1 Demographics

Patient demographics are shown in Table 1. The median age was 20 years [range 7‐38]. The median age of patients treated in pediatric and adult units was 17 [range 7‐19] and 24 [range 18‐38] years, respectively. Beyond treatment location, there were no statistically significant differences between the baseline characteristics of the adolescent population and the comparison young adult group. There was a trend (*P* = 0.05) toward a difference in baseline eGFR, with a difference in median eGFR of 10 mL/min.

**Table 1:** Demographics by age group

	Age group		*P*‐value
	≤18 y (n = 14)	>18 y (n = 19)	
AGE, median (range)	17 (7‐18)	24 (19‐38)	
Treatment location, n (%)			
Children's hospital (DCH)	12 (85.7%)	1 (5.3%)	<0.001
Adult hospital (KCI)	2 (14.3%)	18 (94.7%)	
Sex, n (%)			
Male	8 (57.1%)	13 (68.4%)	0.716
Female	6 (42.9%)	6 (31.6%)	
Race, n (%)			
Caucasian	10 (71.4%)	16 (84.2%)	0.422
Non‐Caucasian	4 (28.6%)	3 (15.8%)	
Ethnicity, n (%)			
Hispanic	12 (85.7%)	17 (89.5%)	1.000
Non‐Hispanic	2 (14.3%)	2 (10.5%)	
Tumor location, n (%)			
Appendicular (extremity, limb girdle)	12 (85.7%)	12 (63.2%)	0.241
Axial (spine, pelvis, head/neck)	2 (14.3%)	7 (36.8%)	
Stage at diagnosis, n (%)			
Localized and resectable	12 (85.7%)	13 (68.4%)	0.416
Unresectable or metastatic	2 (14.3%)	6 (31.6%)	
Initial eGFR, median (range)	114 (87‐157)	124 (96‐144)	0.051
Doses of HDMTX received, n (%)			
12	10 (71.4%)	4 (21.0%)	0.006
<12	4 (28.6%)	15 (79.0%)	
Histologic response, n (%)			
≥90%	7 (50%)	5 (26.3%)	0.218
<90%	7 (50%)	11 (57.9%)	
Never resected	0 (0%)	3 (9.1%)	

### 3.2 Methotrexate clearance

**Figure 2:**
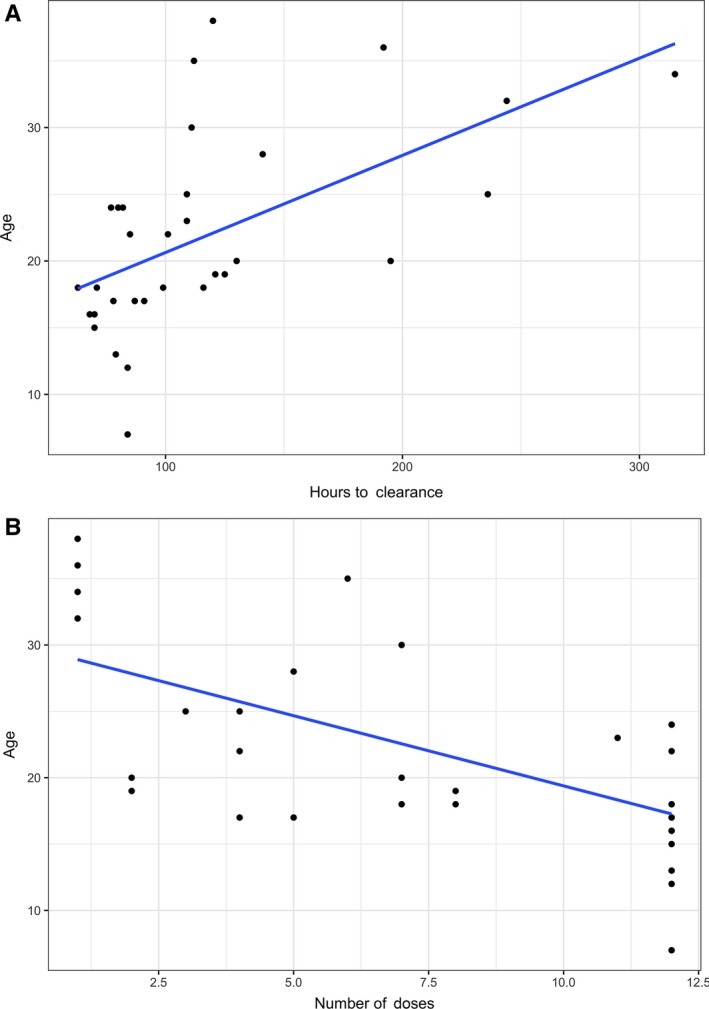
Correlation between age and (A) time to methotrexate clearance (*r* = 0.64, *P* < 0.001) or (B) number of HDMTX doses received (*r* = −0.63, *P* < 0.001)

The median overall clearance time of HDMTX was 99 hours (interquartile range[IQR] 42, range 63‐315). The median clearance time in patients ≤18 years old was 79 hours (IQR 16, range 63‐116), while the median clearance time for patients >18 years old was 120 hours (IRQ 62, range 77‐315; *P* < 0.001). There was a strong positive correlation between age and hours to clearance (Spearman *r* = 0.64, *P* < 0.001; Figure 2A).

### 3.3 HDMTX completion

For patients ≤18 years old, the median number of HDMTX doses received was 12 [range 4‐12] with a median dose of 11.8 g/m^2 ^[range 9.1‐12.3]. Patients >18 years old received a median of five doses of HDMTX [range 1‐12], significantly lower than the younger group (*P* < 0.01) and at a median dose of 10.2 g/m^2 ^[range 6.3‐12.1]. The number of HDMTX doses received was negatively correlated with age (*r* = −0.63, *P* < 0.001; Figure 2B).

Nineteen patients did not receive all 12 planned doses of HDMTX, due to delayed clearance (8), change in treatment plan in reaction to poor histologic response (4); renal insufficiency (4); disease progression (2); or death (1). The one on‐treatment death was in an adolescent who developed sepsis after doxorubicin/cisplatin and was not felt to be related to methotrexate. Of the nineteen who did not receive all 12 doses of HDMTX, 4 received only one dose, and 2 received only two doses. These six patients were all over the age of 18 and experienced delayed clearance. Additional factors contributing to the decision to discontinue methotrexate after only 1‐2 doses in these six patients included the following: renal insufficiency (2) and patient choice (4).

### 3.4 Toxicity

There were no readmissions for methotrexate‐related adverse events and no hospitalizations that were prolonged for toxicity after serum methotrexate clearance. No patients required glucarpidase. Eight patients developed mild renal insufficiency (≤ grade 2 per CTCAE v5) during one or more HDMTX cycles; six of these patients were >18 years old (age 20‐35). No creatinine elevation exceeded 1.7x ULN. Four patients >18 years old who experienced renal insufficiency discontinued treatment for this reason, at least in part. Two patients ≤18 years old experienced renal insufficiency; a 13 year old went on to complete all 12 planned cycles of HDMTX whereas an 18 year old completed 7 of 12 cycles and discontinued due to delayed clearance and patient choice.

There was no age‐related difference in eGFR before the start of chemotherapy compared to after completion of all HDMTX.

### 3.5 Histologic response

Histologic response >90% after neoadjuvant chemotherapy is known to be associated with improved outcomes.^11^ In this cohort, there was no correlation between age and histologic response (*P* = 0.5), but there was a significant positive correlation with the number of HDMTX doses received before resection and histologic response (*r* = 0.49, *P* = 0.006). Patients who received all four planned preoperative doses of HDMTX had significantly higher histologic response (82%) compared to those that did not (49%, *P* = 0.008). The proportion of patients with greater than 90% histologic response was also higher in those who received all four preoperative doses of HDMTX (53%) compared to those that did not (7%, *P* = 0.014).

### 3.6 Metastasis‐free Survival

**Figure 3:**
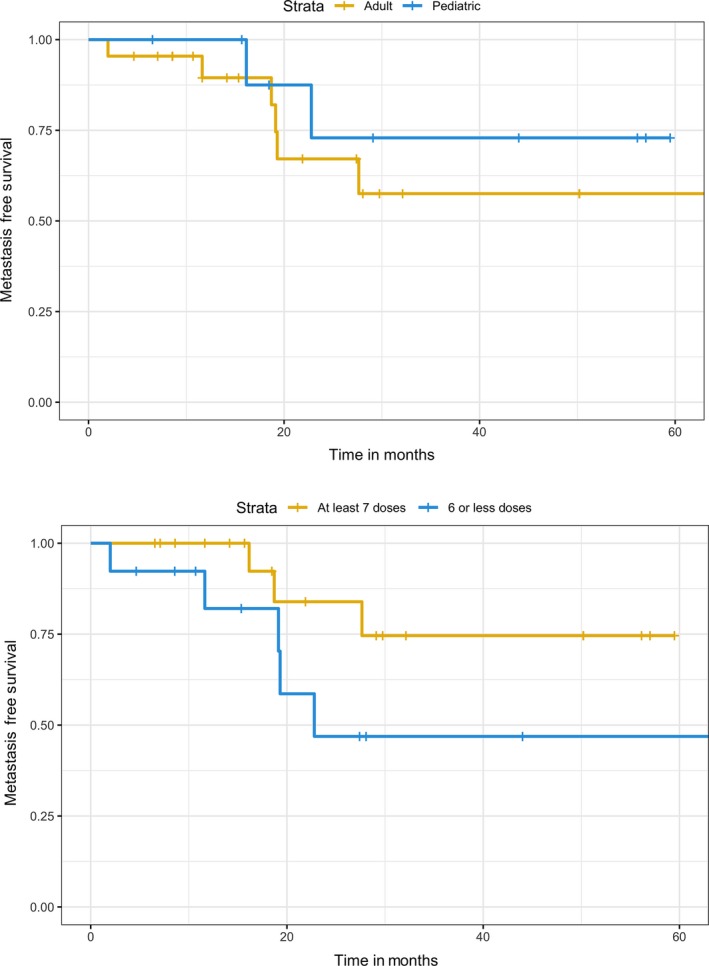
A, In this cohort, there was no significant difference in metastasis‐free survival in young adults (>18 y, n = 13) vs adolescents (≤18 y, n = 12), *P* = 0.47. B, There was a trend toward improved survival among patients who received at least seven doses (n = 17) of HDMTX compared to those who received six or fewer doses (n = 8, *P* = 0.12)

Follow‐up time averaged 27 months (median 21 months, range 2‐64). There was no significant difference in metastasis‐free survival between age groups (Figure 3A). A trend toward metastasis‐free survival was noted for patients who received at least seven doses of HDMTX regardless of age (*P* = 0.12, Figure 3B).

Local recurrence occurred in five patients (15%, ages 15‐35 years), whereas distant metastases developed in six patients (18%, ages 15‐35 years). Two patients with local recurrence are alive with no evidence of disease. Overall mortality rate for the study period was 12% (4 of 33 patients, including one treatment‐related death, ages 15‐28).

## 4 DISCUSSION

The incidence of osteosarcoma has a bimodal age distribution, and poor outcomes among older adults with osteosarcoma are well documented.^1^, ^11^, ^12^ However, even young adults with osteosarcoma have higher rates of relapse than adolescents.^2^ The factors contributing to poorer outcomes in the young adult population are largely unknown, but likely include delayed diagnosis, tumor biology, drug metabolism, and differences in therapy received. Exposure to HDMTX may potentially be a contributing factor.

In this study, we confirmed that young adult patients (ages 19‐38 years) are more likely to experience a delay in methotrexate clearance when compared to adolescents (ages 7‐18 years) despite administration of similar supportive care. Methotrexate clearance time ranged from 3 days to nearly 2 weeks, with a strong correlation between methotrexate clearance and age.

Nonetheless, there were no methotrexate‐related grade 3 or greater toxicities among any patients treated with HDMTX regardless of age. Lack of serious toxicity suggests that with appropriate supportive care, HDMTX can be safely administered to patients over 18 years of age. This is in contradiction to a recent Italian retrospective study of 10 patients ages 16‐23 who received HDMTX.^13^ However, 2 of these 10 patients experienced therapy‐related death suggesting a highly atypical study group. The EUROpean Bone Over 40 Sarcoma Study (EURO‐BOSS)^14^ prospectively gathered data on high‐grade osteosarcoma patients ages 41‐65, an older population than studied here. EURO‐BOSS patients received investigator's choice therapy and included 48 patients who received HDMTX. Among these older patients, 23% experienced delayed methotrexate clearance and only one developed nephrotoxicity ≥grade 2.

Eight patients included in our analysis discontinued HDMTX treatment for a primary reason of delayed clearance. Within the limitations of this retrospective study, we cannot fully determine reasons for early treatment discontinuation in the absence of severe toxicity. In our experience, recurrent and/or significantly delayed clearance not uncommonly results in discontinuation of methotrexate therapy, particularly in young adults who may experience intolerable psychosocial and financial impacts from prolonged hospitalizations. Subsequent delays in doxorubicin/cisplatin therapy due to slow methotrexate clearance may arguably have a negative impact on disease outcomes as well and may play a role in physician decision making in these situations.

We assessed the disease outcomes of histologic response and metastasis‐free survival. Histologic response is a well‐established prognostic indicator in osteosarcoma, and in this study, histologic response was positively correlated with the number of preoperative HDMTX doses received. This finding is further notable since we identified a trend toward improved metastasis‐free survival in patients who received at least seven doses of HDMTX, even among young adult patients with higher risk of relapse based on age. Notably, the reason six patients did not receive all 12 planned doses of HDMTX was due to a change in therapy related to other high‐risk features (disease progression on therapy or poor histologic response). Nonetheless, these results suggest the efficacy of HDMTX for osteosarcoma may extend to patients over the age of 18, when administered in real‐life, non‐trial settings.

Within the limitations of this small, retrospective, single‐institution study, we conclude that HDMTX in young adults with osteosarcoma appears to be effective with low toxicity risks. Increased knowledge of the safety of well‐managed HDMTX such as reported here and by the EURO‐BOSS group may lead to more frequent utilization, which in turn may improve patient outcomes. Patients should be counseled on expected prolonged clearance time, but treatment of HDMTX may be attempted in most young adults with osteosarcoma.

## CONFLICT OF INTEREST

None declared.
